# Geographical Origin Authentication of Agri-Food Products: A Review

**DOI:** 10.3390/foods9040489

**Published:** 2020-04-13

**Authors:** Katerina Katerinopoulou, Achilleas Kontogeorgos, Constantinos E. Salmas, Angelos Patakas, Athanasios Ladavos

**Affiliations:** 1 Department of Business Administration of Food and Agricultural Enterprises, University of Patras, 30100 Agrinio, Greece; akaterin@upatras.gr (K.K.); akontoge@upatras.gr (A.K.); apatakas@upatras.gr (A.P.); 2 Department of Materials Science and Engineering, University of Ioannina, 45110 Ioannina, Greece; ksalmas@uoi.gr

**Keywords:** geographical origin authentication, certification of agricultural products, IRMS, isotope ratio, ICP-MS

## Abstract

This study is a systematic literature review of geographical origin authentication by elemental analytical techniques. Authentication and certification of geographic origin of agri-food products is a useful tool toward the protection of the quality for products. The aim of this work was to map the current state of research in the area of agricultural products and food, identifying emerging fields to the geographical origin of products. The article is divided in three parts. The first part of the article deals with the analytical techniques applied in the food authentication. Special mention is made to elemental analysis and multiple isotope ratio. The second section focuses on statistically published data concerning published research for geographical origin authentication for the period 2015–2019. Specific results are presented inter alia: number of articles according to the type of product, articles according to the type of the analytical techniques, and others. The third part contains characteristic results from articles that were published in the period 2015–2019, on certification of geographical origin on specific agricultural products.

## 1. Introduction

The spread of agricultural products and their appearance on the market as Protected Designation of Origin (PDO), Products Geographical Indication (PGI), traditional specialty guaranteed (TSG), or organic products has attracted considerable attention as an important factor affecting consumer behavior. However, the preservation and strengthening of such production requires quality products, aside from taking the emergence of initiatives to protect them from unfair competition. The most common case is the problem of uncontrolled imported qualitatively inferior agricultural products, which are marketed as ‘local’ or emanating from specific geographic regions. The most common type of fraud in foods, referred to in 95% of publications is the replacement of a component with a similar cheaper component [1], which is difficult to recognize by the consumer and difficult to identify by analytical techniques. Other significant frauds have been found in food of animal origin such as milk and dairy products, honey, fish, and seafood [2]. In several products, undeclared ingredients and genetically modified ingredients have been detected. Since the beginning of 1980s, worldwide research effort to develop new techniques and methodologies has been aimed at targeting and identifying the geographical origin of agri-food products [3].

It is obvious that successful geographical certification could offer many social and economic benefits to producing countries. However, the value and benefits of geographical certification is dependent on the way that an agri-food product is exploited, policed, and marketed.

The utilization of a geographical certification needs to be accompanied by appropriate marketing strategies to develop consumer perception of the superior quality, associated with the geographical origin, and to ensure consumer loyalty.

Food safety is generally controlled with official methods by determining physicochemical or biological parameters in accordance with established specifications and tolerances. Analytical tests for authentication purposes such as recognition of the detection of adulteration or geographic origin are more complex. Specifications for agri-food products with geographic indication are usually based on subjective characteristics such as organoleptic properties. Physicochemical parameters do not allow differentiation from similar products and are generally not characteristic of the geographic origin. Therefore, adulterations or imitation products are offered to consumers and compete in an unfair manner with the genuine product. Amongst the various proposed methods, the isotopic ones have been shown to be an efficient tool against the adulteration of agri-food products, since the beginning of the 2990s after the European Council decision to create an official European isotopic wine databank for the wine sector controls (EC Reg. No. 2048/89). Many European funded research projects have further developed various spectroscopic fingerprinting methods and these isotopic techniques on a wide range of agri-food products to investigate their application in the control of animal, botanical, and geographic origin.

A common subject of physicochemical parameters on agri-food authentication is the need for a databank of genuine samples to which the ‘suspect’ test sample can be compared to establish its authenticity. In order to suggest an authenticity parameter such as geographical origin, a large number of independent parameters is needed to be measured and statistically processed in order to identify key tracers that differentiate the regions or countries of interest.

It is obvious that the authenticity and certification of geographic origin of agricultural products is a useful tool toward the protection of high-quality products. This shift in how consumers share information, gain knowledge, and finally make decisions present significant opportunities for professionals to enhance the effectiveness of the certification of their products. Due to this uniqueness, farm products should have their own identity. In a rapidly moving field such as communications via the Internet and e-marketing, is important for scientists and farmers to monitor and be aware of this. Therefore, the purpose of this work was to map the current state of research in this area, identifying emerging fields to the geographical origin of agri-food products.

## 2. Methodology

In order to provide a comprehensive update on the research situation of the geographical origin, this study employed a systematic literature review (SLR) for the identification, classification, and synthesis of the literature. SLR [4] is a “systematic”, explicit, and comprehensive process for identifying and evaluating the recorded works produced by researchers, scholars, and professionals. Systematic literature review is a review method by employing a series of steps to ensure adequate rigidity and transparency in the process [5]. A key advantage of this “basic scientific activity” [6] is that the researcher understands the scope of the research and the theoretical background [5], ensuring a thorough and impartial understanding in a particular field. Although SLR is more common in medical sciences, nowadays, researchers have indicated the need for a systematic approach to all areas of research [4,6] to ensure full understanding of the level of the previous research and identify areas that need further research. There are nine steps involved in a SLR, starting from the determination of the purpose and objectives of the revision [4,6]. This literature search was conducted using search terms on ‘Elsevier’, ‘Science Direct’, and ‘Scopus’ to find papers published the last five years (2015–2019) that were relevant to the literature review. The search included the term ‘geographical origin authentication’. The number of articles initially identified as potentially important in the current study was quite large. However, when we implemented certain exclusion criteria, the number of articles was reduced considerably.

## 3. Descriptive Findings

The following findings were based on several articles related to the geographical origin of products. The aim in the presentation of descriptive findings was to map the development of the research area for analytical techniques and for products that dominate how their geographical origin is controlled. In recent years, the increased research follows a clear positive trend in the total number of publications and shows a growing interest in the subject [7].

The first part of the work deals with the analytical techniques applied in agri-food authentication. The second section focuses on statistically published data concerning published research for geographical origin authentication for the period 2015–2019 and included the top 10 countries that have published on geographical origin authentication and documents by subject area on geographical origin authentication and preferred journals used for dissemination. Finally, 155 articles were categorized according to the type of product that has been studied and according to the type of analytical technique applied for the authenticity assessment. The third part deals with the characteristic results obtained on specific food and agricultural products such as meat, dairy products, cereals, honey, etc.

## 4. Techniques, Statistically Research, and Applicability of Research

### 4.1. Analytical Techniques Applied in Food Authentication

Nowadays, there is a global need for the assurance of the geographical origin, authenticity, and traceability of food and agricultural products in general. This is due to the increased concern of consumers about food authenticity. The expanded global markets have imposed some assiduous controls on food labelling due to the large amount of illegal profit. In 2000, the EU established traceability as one of the basic tools for consumer protection from adulteration. A continuous interest in avoiding the adulteration of foods and the detailed description and labelling of agricultural products origin has been raised from all parts: traders, producers, and consumers. The large number of European Union members and the vast European area underlie the risks for fraud that could be committed on food-trade (e.g., via transportation means). This new situation has brought about the need to use guaranteed techniques for the accurate definition of the geographical origin of products. These techniques may already be tested for other purposes.

Modern instruments used for the above processes include plasma laser induced breakdown spectroscopy (LIBS) [8], fourier-transform infrared spectroscopy (FTIR), near-infrared (NIR) and mid-infrared (MIR) spectroscopy, chromatographic techniques as gas chromatography–mass spectrometry (GC-MS) or high-performance liquid chromatography (HPLC), nuclear magnetic resonance (NMR), electron spin resonance (ESR), polymerase chain reaction (PCR), enzymatic assays (ELISA), techniques DNA based and isotope-ratio mass spectrometry (IRMS) with high measurement accuracy, which were developed to serve this need [3,9,10,11].

The organic components of food are dependent on various conditions (e.g., fertilization, botanical origin, history of the field, climatic conditions in the year of cultivation, location, and composition of the soil), whereby it is not always possible to determine the origin of the product by analysis of the organic components. Despite uncertainty about the organic compounds in a sample, the content of selected elements (trace elements and rare earth elements) in agricultural products reflects the growth conditions in the particular environment. Furthermore, the isotope ratios (e.g., carbon, hydrogen, nitrogen, sulfur, strontium, and lead) can provide uniquely representative fingerprints, enabling discrimination of the origin of food samples [12]. In recent years, with the development of new advanced analytical techniques (e.g., thermal ionization MS (TIMS), mass spectrometry inductively coupled plasma multiple collectors (MC-ICP-MS), dynamic mass spectrometry plasma inductively beams (DRC-ICP-MS)), stoichiometric and isotopic composition of any food sample can be obtained and the geographical origin successfully identified.

IRMS has been recognized as a promising procedure that could improve the traceability of plant/animal derived food-products [13]. This method involves the bio-elements in the traceability approaches. Bio-elements are chemical elements that are found in the molecules and compounds that make-up a living organism. In decreasing order, the appearance of the most common bio-elements in a living organism body are oxygen (O), carbon (C), hydrogen (H), nitrogen (N), calcium (Ca) phosphorous (P), sulfur (S), potassium (K), and magnesium (Mg). Ninety-nine percent of body mass consists of the first six bio-elements and 1% of the rest. Measurements of isotope ratio of some light bio-elements have been carried out for various products like oils, alcohol drinks, rice, orange juice, meat, milk, etc. These light bio-elements (C, H, N, O, and S) are used for the development of anti-fraud methods that are based on the stable isotope ratio. The IRMS technique has gained growing interest in the study of the geographical origin of agri-food products, giving the possibility of testing food quality and uniqueness, particularly in cases where conventional analytical methods cannot provide reliable results.

#### 4.1.1. Elemental Analysis

Multilevel composition of animal tissues reflects to extent of the germination animals eat. The vegetation reflects the place where the soil composition developed. The availability of the trace elements depends on various factors (e.g., soil pH, moisture, clay and other characteristics). Accordingly, bioavailable nutrients found beneath the soil can give us direct information on the geographical origin of food and agricultural products.

The techniques used for the elemental footprint of food and drinks are mostly those with multiple sensing elements [12]. Inductively coupled plasma (ICP), in conjunction with atomic emission spectroscopy (ICP-AES), provides high reproducibility and a quantitative linear range that are better than conventional methods with arc or spark, enabling the parallel determination of non-metals and metals. ICP-AES utilizes a high-temperature excitation source and molecular interferences are significantly reduced. The analysis is accurate and the samples must be introduced in dissolved form. The atomic mass spectroscopy, inductively coupled plasma ICP-MS is a powerful tool for elemental analysis, yielding a threshold in a plurality of detectable elements (LOD) for more than 70 elements in low concentrations, (ppb or ppt).

Atomic absorption spectroscopy (AAS) is also a technology used for controlling food identity. AAS spectrometers can analyze only one or a few items at once. Flame AAS (FAAS) has the advantages of the low operating cost and good analytical performance. The major disadvantage of AAS spectroscopy is the lack of multi-component analysis.

The method of neutron activation analysis (NAA) is significant for multi-component analysis, especially for difficult dissolved solid materials. NAA allows discrete sampling of elements as it disregards the chemical form of a sample, and focuses on its nucleus. A sample is subjected to neutron flux produced radioactive nuclides and when these nuclides disintegrate, they emit gamma rays, whose energy properties are characteristic of each nuclide. Comparison of the intensity of the ray with those emitted from a template allows for the estimation of the concentrations of the various nuclides. The major limitation of this technique is the difficulty in analyzing elements (e.g., Sr, Nb, Y, and others with geological interest), while a particular advantage is that it does not destroy the sample.

Particle-induced x-ray emission or proton-induced x-ray emission (PIXE) is a technique used to determine the elemental composition of a material. When a material is exposed to an ion beam, it shows individual interactions that emit electromagnetic radiation with a wavelength in the spectrum of x-rays specific for each element. PIXE is a non-destructive analysis technique that is capable of detecting origin, dating, and authenticity of various products.

The method of x-ray fluorescence spectroscopy (XRF) detects fluorescence generated from the sample excited by high-energy x-rays. XRF offers the possibility of multi-component analysis in solid samples in a wide dynamic range. The main problems of XRF are limited sensitivity for high mass elements and the necessity for pure samples. Even with the use of ultrapure reagent, impurities can appear, therefore it is mandatory to carefully measure the reference samples.

#### 4.1.2. Multiple Isotope Ratio

Based on their atomic mass, the stable isotopes are divided into:

(A) Light isotopes. For the identification of food authenticity, the ratios tested were mostly ^13^C/^12^C, ^15^N/^14^N, ^18^O/^16^O, ^2^H/^1^H, and ^34^S/^32^S (rarely).

(B) Heavy isotopes. The most common are ^87^Sr/^86^Sr and rarely ^206^Pb/^204^Pb, ^207^Pb/^204^Pb, ^208^Pb/^204^Pb, and ^143^Nd/^144^Nd.

The ratios of the stable isotopes above-mentioned were analyzed reliably and accurately by the technique of the differential analysis of the samples and standards. The isotope ratio (IR) of an element X is the ratio of the concentration of the heaviest isotope (rare) to the concentration of the lighter (common) present in a sample. The isotopic abundance of a sample relative to a reference sample is expressed by the term R_sample_ in Equation (1):

(1) $$R_{sample}=\frac{{}^{H}X}{{}^{L}X}$$

The R_standar__d_ is the isotope ratio of the chemical element (X = H, C, N, O, or S) for certain materials that were selected from scientists as international standard materials. Factor δ uses the fraction R_sample_ over R_standard_ multiplied by 1000, aiming to achieve big enough delta values that could show the differences in isotope concentration. The equation that expresses the delta notation (δ) is the following:

(2) $$\delta =\left(\frac{R_{sample}}{R_{standard}}\;\;-\;\;1\right)*1000$$

Isotopic ratios of ^13^C/^12^C, ^15^N/^14^N, ^18^O/^16^O, ^2^H/^1^H, and ^34^S/^32^S are usually analyzed by isotope ratio MS (IRMS). For the determination of isotope ratios, the analytes must be converted to the gas phase—H_2_, N_2_, CO, CO_2_, and SO_2_—before ionization.

For heavier elements (e.g., Pb and Sr), the stable isotope ratios substantially depend on the origin of the test product and can be used to identify the origin of the product. Strontium is commonly used for heavy metal isotope ratio analysis. For measurement of the Sr isotope, Pb and other heavy isotopes is most appropriate to use with other techniques (e.g., TIMS, DRC-ICP-MS, and MC-ICP-MS). The disadvantages of the above MS techniques are the high operating cost and the need for trained personnel, and the advantage is that the isotopic composition of the samples is determined with reliability and precision.

### 4.2. Statistically Research for Published Data Concerning Geographical Origin Authentication for Years 2015–2019

**Figure 1:**
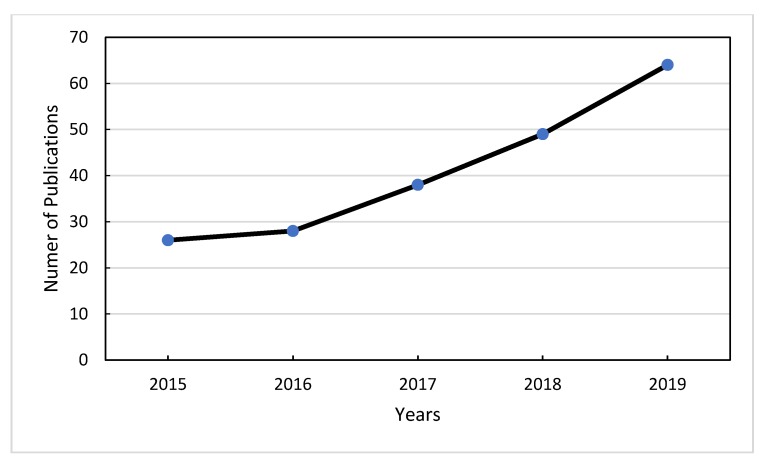
Published research for geographical origin authentication in the last five years.

A total of 75.6% of the publications on geographical origin authentication are original works published in research journals. Review and conference proceedings articles accounted for up to 18% and 6.4% corresponded to book chapters/books. Authentication of geographical origin, fraud, mislabeling, and food safety were amongst the main aspects in food control. During this search, 78 articles were published during 2010–2014 and 205 articles during the period 2015–2019. Published research during the period 2015–2019 is depicted in Figure 1 and shows that the scientific interest in geographical origin authentication has constantly increased.

**Figure 2:**
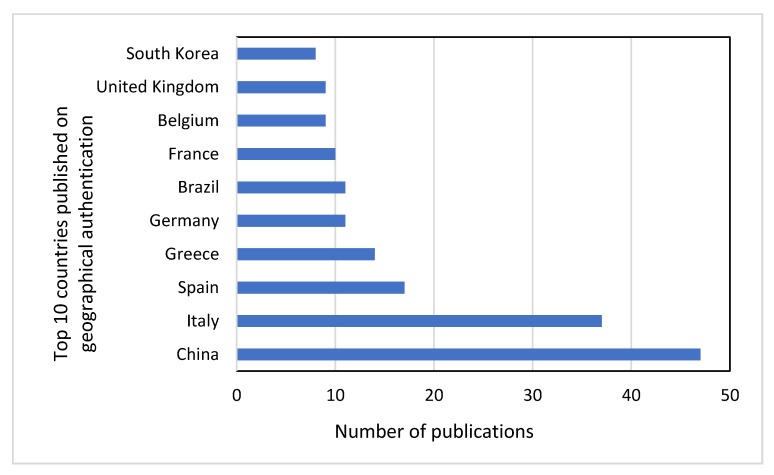
Origin countries of publications on geographical origin authentication.

China is an emerging country in the field, showing a rapid growth of publications during the last five years (Figure 2). It is worth noting that in the top 10 countries list, only three are non-European. This indicates the interest of Europeans in geographical origin authentication, which is supported by national and EU legislation in European countries. Interest in Europe concerning food authentication was also shown in a review paper by Danezis et al. [7], where it was published that eight European countries out of 10 countries in total, were involved in food authentication studies until 2014. Italy was the first country in publications during the same period, while China was in seventh position [7].

**Figure 3:**
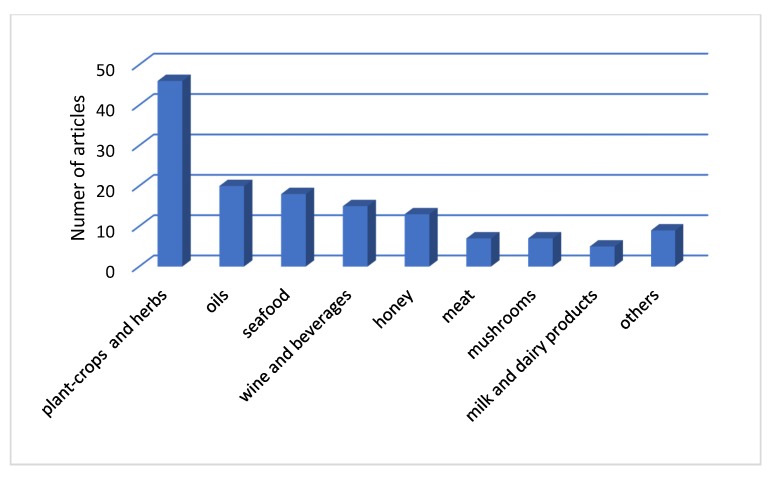
Number of articles according to the type of product.

**Figure 4:**
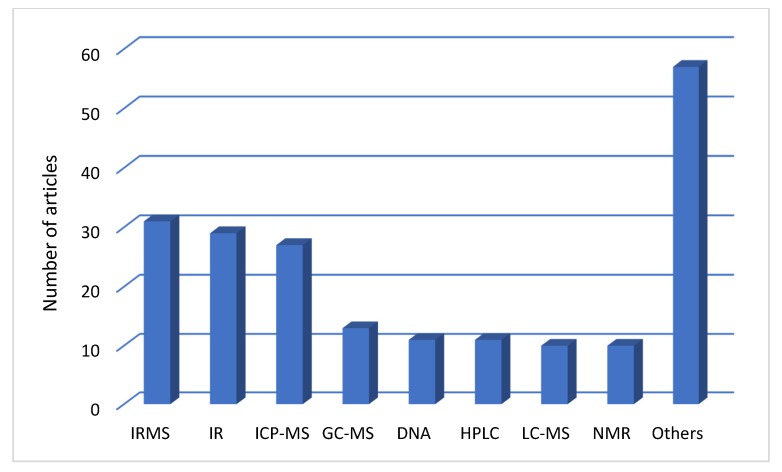
Articles according to the type the analytical techniques.

From the 205 documents that were published during the period 2015–2019 in the area of geographical origin authentication, 155 were articles and the others were review papers, books or book chapters, and articles on conference proceedings. These 155 articles were categorized according to the type of product that had been studied (Figure 3) and the type of the analytical technique that was used for the authenticity assessment (Figure 4).

Figure 3 depicts the number of articles according to the type of products that have been studied including seafood, meat, milk and dairy products, wine and beverage products, plant-crops and herbs, honey, oils, mushrooms, and others (e.g., eggs, pepper, maize).

The second categorization was made according to the type of the analytical technique that was used for the authenticity assessment including IRMS, ICP-MS, NMR, GC-MS, HPLC, LC-MS, IR, DNA, and others (e.g., HRMS, Raman, ICP-OES, SIFT-MS etc.). In many articles, we came up with more than one technique for studying each product, which is the reason why there is a large number of ‘other’ techniques in Figure 4.

As shown in Figure 4, IRMS, IR, and ICP-MS were the three techniques that were most preferred for geographical origin authentication. In particular, IRMS and ICP-MS were combined for the analysis of many products in many articles.

### 4.3. Published Research for Geographical Origin Authentication from 2015 to 2019

Tasks relating to the implementation of techniques for identifying the geographical origin were found that dealt with a variety of products. Some articles, according to the type of product, are referenced below. In the category of ‘plant-crops and herbs’: potato [14], vegetables: lettuce, pepper, tomato [15], onions [16], rice, flour and cereals [17], oranges [18], herbs [19,20,21]. In the category of ‘oils’: olive oil [22,23,24]. Other categories of ‘seafood’: scallops [25], eel [26], sea bass [27], clams [28], ‘honey’: thyme honeys [29], pine, and thyme honeys [30], commercial honey [31], [32] ‘meat’: [33], lamb (South Africa) [34], ‘milk and dairy products’: milk and cheese from buffalo [35], sheep milk [36] and others [37].

More specific, in the category of ‘plant-crops and herbs’, which is the category with the largest number of publications, there were articles with various analytical techniques for geographical origin authentication. The article by Wu et al. [17] contained a study of the geographical origin of cereal grains by mass spectrometry of the stable isotope element (EA-SIRMS). Set stable isotope of carbon and nitrogen compositions (δ^13^C, δ^15^N) for different cereal species from various cultivated areas (USA, Canada, Australia, and some provinces of China) such as rice, wheat, maize etc., it was found that values of δ^13^C showed significant variation for different cereal grains and origins. No significant variation of δ ^15^N values were observed. Moreover, they refer to the fact that there is little information on the annual changes in the isotopic composition of rice, wheat, and even whole grain. Therefore, further statistical investigation based on a large number of samples is required in order for the method to become a strong tool to discriminate geographical origin.

**Figure 5:**
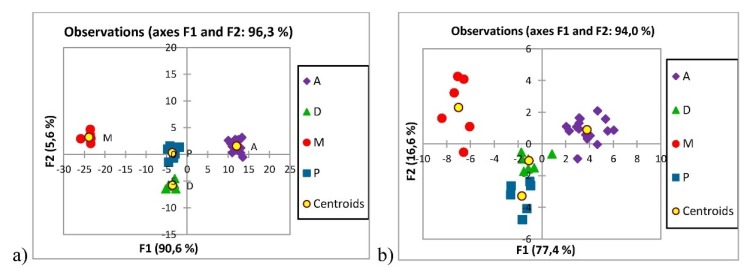
Discriminant score plots (**a**) of the 36 Slovenian potato samples from the Alpine (A; *n* = 16), Dinaric (D; *n* = 8), Mediterranean (M; *n* = 6), and Pannonian (P; *n* = 6) regions (**b**) with the rare earth elements excluded. The centroids represent the means of each group (Source: [38]).

Mahne Opatić et al. [38] studied the geographical origin of organic cultivated potatoes (Solanum tuberosum L.) from four regions of Slovenia: Alpine, Dinaric, Mediterranean, and Pannonian macro-regions. Samples were subjected to stable isotope analysis of the major bio-elements (δ^13^C, δ^15^Ν, δ^18^O, δ^34^S) and to element profiling (Na, Mg, P, S, Cl, K, Ca, Mn, Fe, Ni, Cu, Zn, Mo, Br, Rb, Sr), which included the rare earth elements (Sc, Y, Nb, La, Ce, Pr, Nd, Dy, Er). The classification was successful among the four groups of the Alpine, Dinaric, Mediterranean and Pannonian macro-regions, with an overall success rate of 100% obtained. In particular, the most influential parameters in the proposed model were the rare earth elements. Figure 5 shows the discriminant plots for the first two functions obtained by the discriminant analysis of these Slovenian potato samples. The potatoes were successfully separated into four groups based on their geographical origins. The first discriminant function (F1) explained 90.6% of the total variance, while F2 explained 5.6%. The model was validated according to the prediction ability determined by cross-validation, which achieved a prediction rate of 100.0%.

Therefore, the tested parameters and the proposed methodology could provide traceability for the Slovenian potatoes nationwide. Moreover, this excellent predictive ability of the proposed model on a smaller scale (i.e., national) is very promising for further studies on larger scales (i.e., international). The proposed methodology could be applied to existing food regulations and trade agreements.

The discrimination for samples of lettuce, sweet peppers, and tomatoes depending on their country of origin (Slovenia, Austria, Spain, Morocco) was obtained by Mahne Opatić et al. [15], with application of IRMS and elemental composition analysis. Although there was a slight overlap of the measured parameters among some samples, definite tendencies of these samples toward different countries of origin were observed. The IRMS method, in combination with elemental composition parameters and supervised pattern-recognition multivariate technique (i.e., the DA classification), represent useful and powerful implements for rapid and robust screening of extensive numbers of samples.

**Figure 6:**
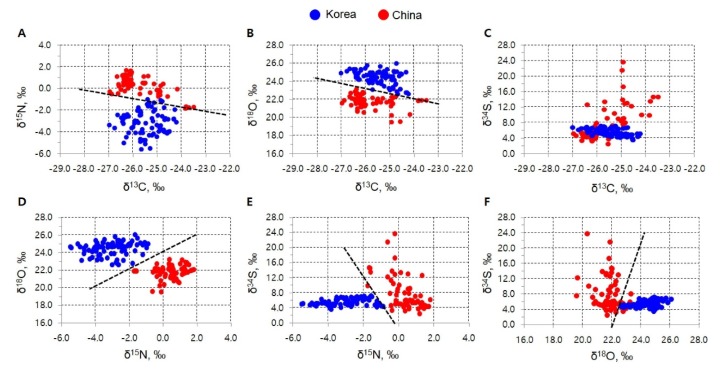
2D plots of (**A**) δ^13^C vs. δ^15^N, (**B**) δ^13^C vs. δ^18^O, (**C**) δ^13^C vs. δ^34^S, (**D**) δ^15^N vs. δ^18^O, (**E**) δ^15^N vs. δ^34^S, and (**F**) δ^18^O vs. δ^34^S values of the sliced shiitake mushrooms from Korea (blue) and China (red) (Source: [39]).

The study by Chung et.al. [39] extends the knowledge of geographical differences between China and Korea, evidenced by the shiitake (mushroom) isotope fingerprint. Stable isotope ratios (δ^13^C, δ^15^N, δ^18^O, and δ^34^S) were measured with a stable isotope ratio mass spectrometer, and a geographical discrimination method using orthogonal projection to latent structure-discriminant analysis was developed. All of the 2D plots, except for those of δ^34^S versus δ^13^C (Figure 6C), showed clear separations between Korean and Chinese samples. In particular, the δ^15^N and δ^18^O values were critical isotope markers for an obvious geographical origin discrimination between mushrooms produced in Korea and China, as shown in Figure 6.

Ratios of stable isotopes of carbon (C), hydrogen (H), oxygen (O), nitrogen (N), and sulfur (S) were used for the evaluation of the geographical origin of onion cultivated in various regions of Korea by Park et al. [16]. They concluded that this technique has high potential for discriminating onions produced in Korea from those produced in other countries and more distinct geographical differentiation of onion origin could be achieved by additional multi-elemental analysis.

Apart from IRMS and ICP-MS, there were other publications that suggest that IR and chemometrics can be used for the detection of the origin of agri-food products [20,21,40,41,42,43,44,45,46].

Teye et al. [47] suggested that NIR spectroscopy, in combination with the appropriate chemometrics, could be used for rapid and nondestructive detection of rice authenticity and quality. The systematic selection of different preprocessing methods (MC, DT, SNV, and MSC) with principal component analysis (PCA) and modeling with the k-nearest neighbor (KNN) and support vector machine (SVM) multivariate calibration model showed that MSC + PCA plus KNN showed superiority in this study, with more than a 90% classification rate for all categories of rice samples studied.

Additionally, Kahmann et al. [48] tried to classify a herb called ‘yerba mate’, according to geographical origin. For that matter, NIR spectroscopy and element concentration data were employed to describe 54 samples from four South American countries. Three classification techniques were tested: k-nearest neighbor (KNN), support vector machine (SVM), and discriminant analysis (DA). SVM was able to correctly classify all samples from the training set in both datasets; as for the testing set, it correctly classified 94.29% in the NIR data and 100% in the elements data. When compared to other variable selection techniques, their propositions proved to be more robust by providing a better classification in both datasets.

A large number of publications referred to ‘oils’ and especially to olive oil. These articles were published from research groups from Mediterranean countries such as Spain, Italy, Turkey, Cyprus, Tunisia, and Morocco. The remaining papers concerned palm oil and patchouli oil. Techniques used to certify geographical origin were mainly HPLC, IRMS, and MS (ICP-MS, GC-MS, LC-MS). Determination of indicators for geographical origin is complicated, since chemical composition of virgin olive oil is influenced by various factors such as botanical origin, climatic conditions, soil, degree of ripening of olives, and the method of extraction. Authenticity studies have been reported for the classification of olive oils according to their botanical or geographical origin based on determinations of variable counts for their major or minor constituents such as fatty acids profile of olive oils, phytosterols, phenols, squalene, volatiles, or a combination of two or more components [22,23,49,50].

Kritioti et al. [23] studied two dominant Cypriot olive cultivars, Cypriot (ladoelia) and Koroneiki (lianolia). Their oils were analyzed for fatty acid composition by GC/FID. Virgin olive oils, VOOs, were differentiated mainly due to the variety, Koroneiki or Cypriot, based on the fatty acids composition. Across all districts, VOOs from the Cypriot cultivar are characterized by higher concentrations of saturated fatty acids (SFA )and polyunsaturated fatty acids (PUFA) compared to those from Koroneiki. VOOs from the Koroneiki variety possess higher concentrations of monounsaturated fatty acids (MUFA), and ω-9 fatty acids, when compared to oils of the Cypriot cultivar, thus exhibiting high oxidative stability. Geographical discrimination, through altitude and district factors, was not satisfactory, mainly due to the different parameters such as rain-fed/irrigation systems and degree of olive ripeness throughout the four districts, the three altitude zones, and the small land size of the country.

In the study of Gumus et al. [24], trace element contents and stable carbon isotope ratios of olive oils were determined in order to evaluate their potential as authentication parameters. Forty-nine virgin olive oil samples (VOOs) from six different areas of the western part of Turkey were analyzed with ICP-MS and EA-IRMS. The geographical origin of the olive oils could be successfully discriminated with trace element content in combination with IRMS analysis. The results showed that there is significant potential for successful authentication of olive oils from Manisa, based on Fe and Zn content, and δ^13^C have a dominant effect on the discrimination of olive oils originated from Edremit Bay. The outcome of this study could be used to identify the geographical origin of olive oils, especially for Manisa and Edremit Bay areas.

The third large category of publications concerned ‘seafood’, namely scallops, clams, eel, sea bass, salmon, shrimps, and sea cucumber. Twenty-six wild-type salmon fished in Canada and a total of 74 farmed salmon were considered in the study by Fiorino et al. [51]. The profiling of the 30 major fatty acids in salmon lipid extracts, rapidly provided by DART-HRMS (direct analysis in real time-high resolution mass spectrometry) and then integrated by data elaboration based on principal component analysis (PCA), enabled a fast discrimination between Canadian wild-type salmons and farmed salmons from Canada, Chile, and Norway. Saturated FA (fatty acids) and polyunsaturated FA with 20 or 22 carbon atoms on their side chain including the omega-3 species EPA and DHA, were found to be more abundant in wild-type salmons, whereas unsaturated FA like 18:1, 18:2, and 18:3 and several oxidized derivatives of the latter appeared to be more relevant in farmed salmons. Relative abundances of DART-HRMS signals related to specific FA appear to be very promising for the differentiation of wild-type salmon from farmed ones, a very relevant issue in the context of consumer protection from seafood fraud.

**Figure 7:**
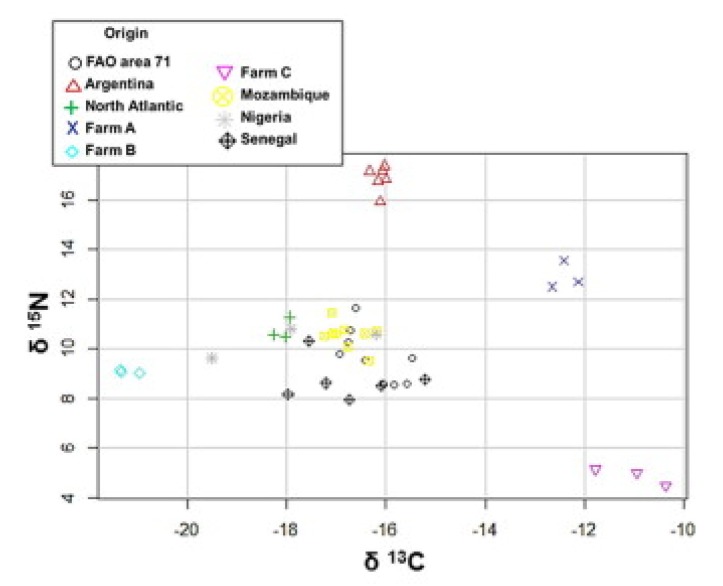
Scatterplots of C and N isotope ratio analysis for origin assessment (Source: [52]).

The aim of the study by Ortea and Gallardo [52] was to determine whether shrimp species, geographical origin, and production method (wild vs. farmed) could be differentiated using either SIR analysis of carbon and nitrogen or multi-element composition (Pb, Cd, As, P, S) or a combination of both. Samples were analyzed using an isotope ratio mass spectrometer, with inductively coupled plasma-optical emission spectrometry (ICP-OES) for P and S, and an inductively coupled plasma-mass spectrometer (ICP-MS) for As, Cd, and Pb. For origin assessment, a total of nine different groups were considered, namely, FAO area 71, Argentina, North Atlantic, Mozambique, Nigeria, Senegal, and farms A, B, and C. The results that concerned geographical origin assessment are depicted in Figure 7.

Geographical origin of 34 commercial thyme honeys produced in specific Mediterranean countries (Egypt, Morocco, Greece, and Spain) was investigated by Karabagias et al. [29]. Minerals were quantified using inductively coupled plasma optical emission spectroscopy (ICP-OES). Results of this study showed that: (a) conventional physicochemical and color parameters, (b) mineral content, and (c) the combination of both using chemometrics may successfully differentiate the geographical origin of commercial thyme honey from different countries. In another article, Karabagias et al. [30] investigated pine and thyme honeys produced in Greece. Conventional physicochemical and color parameters were determined using official methods of analysis. Twenty-five minerals were quantified using inductively coupled plasma-atomic emission spectroscopy (ICP-AES). Results showed that pine and thyme honeys recorded variations in their mineral content according to geographical origin, and the results successfully differentiated the geographical origin of each honey type from four different regions in Greece.

Stable isotope ratio (^13^C/^12^C and ^15^N/^14^N) of lamb meat from seven different farms in South Africa were studied by Erasmus et al. [34] in order to assess a tool for authenticating the origin and feed status. They mentioned that the analysis of the lamb stable isotope ratio is an assessment tool to assess the extensive meat production in South Africa associated with diet type, whose isotope ^13^C is particularly useful for displaying feed variations. The results were reasonable and representative of the observed vegetation of different areas. They also referred that through the analysis of additional isotopes, one could even further improve the discriminative power of the technique.

**Figure 8:**
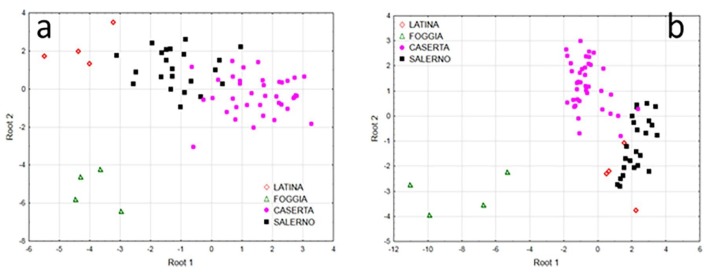
Score plots of canonical discriminant analysis of the isotopic and elemental composition of milk and Mozzarella di Bufala Campana PDO from Caserta, Salerno, Latina and Foggia, for milk (**a**) and mozzarella (**b**) (Source: [35]).

Mozzarella di Bufala Campana (MBC) PDO cheese is produced from whole buffalo milk in certain regions of southern Italy. Bontempo et al. [35] found significant differences between milk and cheese, mainly related to curd formation and brining. Furthermore, the season and production district within the PDO reference area significantly influenced the elemental and isotopic composition of dairy products, interacting with processing variability. Under the specific conditions considered in this study, in which different categories of mozzarella were processed in the same cheese factory, animal husbandry and geographical origin would appear to have a greater effect on the elemental and isotopic fingerprint of cheese than processing (Figure 8). Therefore, the ratio analysis of stable isotopes in combination with elemental analysis is a powerful technique for detecting the authenticity of PDO cheeses.

There have been numerous applications on other types of agricultural and food products such as sea cucumber [25,53], coffee beans [54,55], tea [56], and flour [57,58], demonstrating the importance of the issue and the increased interest of the international scientific community in this.

## 5. Conclusions

Published research during the period 2015–2019 showed that the scientific interest in geographical origin authentication has increased. Therefore, it has become clear that authentication and certification of the geographical origin of products is a useful tool in protecting quality products.

There are many techniques related to the geographical origin of products. Most of them appear very promising for geographical origin authentication, a very relevant issue in the context of consumer protection from fraud. In many articles, IRMS and ICP-MS techniques were combined for the analysis of a variety of products, and from the above information, it is evidenced that their combination can be a powerful tool for geographical origin authentication.
